# Adolescent Victim Types Across the Popularity Status Hierarchy: Differences in Internalizing Symptoms

**DOI:** 10.1007/s10964-021-01498-w

**Published:** 2021-09-28

**Authors:** Sarah T. Malamut, Molly Dawes, Yvonne van den Berg, Tessa A. M. Lansu, David Schwartz, Antonius H. N. Cillessen

**Affiliations:** 1 Behavioural Science Institute, Radboud University, Nijmegen, Netherlands; 2 INVEST Research Flagship Center/Psychology, University of Turku, Turku, Finland; 3 College of Education, University of South Carolina, Columbia, SC USA; 4 Department of Psychology, University of Southern California, Los Angeles, CA USA

**Keywords:** Victimization, Popularity, High-status victims, Internalizing, Person-oriented analyses, Multi-informant

## Abstract

Previous studies have called attention to the fact that popular youth are not immune to peer victimization, suggesting there is heterogeneity in the popularity of victims. Yet, no study to date has determined whether victims with different levels of popularity status can be identified using person-oriented analysis. Such analysis is critically needed to confirm the existence of popular victims. Further, there remains a paucity of research on internalizing indices of such popular victims, especially compared to other victim and non-victim groups. To address this gap in the research literature, the current study used latent profile analysis to identify subgroups of victims based on victimization (self- and peer-report) and popularity (peer-report). This study sought to verify the existence of popular victims and to compare victim subgroups on loneliness and self-esteem. Participants were 804 Dutch adolescents (50.2% boys, *M*_age_ = 13.65 years, ranging from 11.29 to 16.75 years). The results revealed six subgroups, including a group of popular self-identified victims. Popular self-identified victims were generally less lonely than other victims, but had higher loneliness and lower self-esteem than non-victims. Implications are discussed for understanding the victimization experiences of high-status youth.

## Introduction

Peer victimization is associated with a range of social, emotional, behavioral, and academic difficulties for victims (e.g., Moore et al., 2017), the effects of which can last well into adulthood and underscore the need to comprehensively identify those who are most likely to be targeted (e.g., Wolke et al., 2013). However, successful identification of victimized youth is complicated as discrepancies between peer-reports and self-reports of victimization frequently arise (e.g., Graham & Juvonen, 1998; Lansu et al., 2017; Scholte et al., 2013). Futheremore, recent evidence demonstrates heterogeneity among victims in terms of social status, indicating that being victimized does not necessarily denote low social status (Dawes & Malamut, 2018). The notion that some youth are both victimized and popular challenges entrenched beliefs that high levels of popularity insulate youth from being targets of peers’ aggression. Concerningly, teachers and parents alike question whether youth are actually victimized when they do not align with common assumptions about the adjustment profiles of victims (Mishna et al., 2006). Identifying victims with varying levels of popularity will add to the growing empirical evidence that challenges such long-standing and incorrect assumptions about victimized youth, as these false assumptions can be a barrier to providing the necessary supports to victims who do not fit the stereotypical victim mold. To address this societal need and gap in the literature, this study aimed to better understand the status heterogeneity in victimized youth and its implications for adjustment.

### Victim Groups and Popularity

Victimization is commonly assessed using peer- and self-report measures (Graham & Juvonen, 1998; Scholte et al., 2013). By comparing the (lack of) agreement between them, four groups of victims can be identified: (1) convergent victims (high levels of both self- and peer-reported victimization), (2) self-identified victims (high levels of self-reported and low levels of peer-reported victimization), (3) peer-identified victims (low levels of self-reported and high levels of peer-reported victimization), and (4) non-victims (low levels of both self- and peer-reported victimization). However, this previously established classification does not consider differences in social status *across* and *within* these groups which likely exist, given that victimization does not only occur among youth at the bottom of the social hierarchy (Dawes & Malamut, 2018).

Perpetrators may have an incentive to target peers with high status, as popular peers have access to and power over social resources. As aggression can be proactive, strategic, and instrumental behavior (e.g., Faris et al., 2020), some youth may target popular peers as a “high-risk, high-reward” means to take the popular peers’ access to limited social resources for themselves. Relatedly, youth with high status may choose to target other high-status peers in an attempt to protect their own social standing, as they may perceive high-status peers as a potential threat to themselves (i.e., social competitors). Empirical evidence supports these ‘taking resources’ and ‘status protection’ processes, as youth who target high-status peers increased in social network prestige and tended to be high in status themselves (Andrews et al., 2016).

Given the theoretical and empirical reasons why high-status youth are also at risk to be victimized, their victimization experiences are more likely to be identified by self-reports rather than peer-reports. Peer reports capture youth who have a reputation as a victim. Because popular youth have a reputation as powerful and admired, it is unlikely then that peers will see them as victims of aggression. Indeed, correlations between peer-reported popularity and victimization are negative (*rs* = −0.10 to −0.59, e.g., Bouman et al., 2012; de Bruyn et al., 2010; Garandeau et al., 2019). In contrast, self-reported victimization has been found to be positively associated with popularity (Malamut et al., 2020). Thus, it is possible that a subset of self-identified victims includes high-status youth who are not seen as victims by their peers.

### Victim Groups and Adjustment

As victimization is consistently marked by internalizing problems and emotional maladjustment (McDougall & Vaillancourt, 2015), the second aim of this study was to compare victim groups with potentially varying levels of popularity on internalizing symptoms. The current study focused on two indices of internalizing symptoms: peer-related loneliness and self-esteem. Loneliness is typically defined as a negative emotional response to self-perceived deficiencies in relationships (i.e., a mismatch between desired and achieved levels; Peplau & Perlman, 1982). Self-esteem is defined as a person’s view of their own abilities or characteristics. Self-esteem and loneliness are negatively associated (see, for a meta-analysis, Mahon et al., 2006) and both loneliness and low self-esteem in adolescence have important implications for future adjustment that can last into adulthood (e.g., Steiger et al., 2014; von Soest et al., 2020). For instance, peer-related loneliness is associated with rejection and poor friendship quality, self-esteem is a critical factor for youth’s physical and mental health, and both are associated with victimization (Maes et al., 2016; Orth et al., 2008; van Geel et al., 2018).

First, internalizing symptoms were expected to vary *across* victim groups. Victimization is generally a strong predictor of internalizing problems (e.g., Reijntjes et al., 2010). Yet, self-reported victimization appears to be more strongly associated with internalizing problems than peer-reported victimization (e.g., Bouman et al., 2012; Scholte et al., 2013). The subjective experience of being victimized may confer more risk than having a victim reputation (Scholte et al., 2013). This may be due to cognitive processes triggered by victimization such as characterological or behavioral self-blame, in which youth blame their victimization on an aspect of their own character or behavior (Graham & Juvonen, 1998), and negatively biased processing of social situations (Lansu et al., 2017).

Second, the current study examined whether popularity amplified or mitigated differences in internalizing symptoms *within* and *across* victim groups. Recent research suggests that both high- and low-status youth report loneliness (Ferguson & Ryan, 2019). All self-identified victims, irrespective of their social status, may experience internalizing problems. However, the unique co-occurrence of being victimized and being popular may impact the negative consequences of victimization experiences.

On the one hand, high-status youth may experience elevated psychological distress when they are victimized because they have “more to lose” given how integral status is to their identity and self-concept (Faris & Felmlee, 2014, p. 235). On the other hand, popularity may instead dampen the association between victimization and internalizing problems. Being popular is a desirable resource in the peer network (LaFontana & Cillessen, 2010) and may prompt positive appraisals of oneself and one’s social accomplishment, leading to higher self-esteem (Litwack et al., 2012). Likewise, popular youth are socially central and desired as friends (e.g., Logis et al., 2013), and this social capital may buffer the association between victimization and loneliness. To understand the complex associations between popularity, victimization, and internalizing adjustment, the current study examined these competing hypotheses.

## Current Study

The goal of this study was to examine the status heterogeneity of victimized youth and its implications for loneliness and self-esteem. To fill these gaps, the current study used person-centered analyses to identify victim types (based on self- and peer- reports of victimization) varying in popularity, and examined differences in loneliness and self-esteem between the identified types. Variation in popularity was expected for self-identified victims, but not for peer-identified victims or convergent victims. The peer-identified and convergent subgroups were expected to be homogenous in terms of low popularity, given that these groups are known to experience social difficulties and are seen as unpopular, rejected, and low in number of friends.

In addition, the victim types were compared on loneliness and self-esteem. Self-identified victims were expected to report higher levels of loneliness and lower self-esteem than peer-identified victims and non-victims. Convergent victims were expected to be most lonely and have the lowest self-esteem because they both see themselves as victims and are seen as victims by their peers. Finally, it was examined whether popularity amplified or mitigated the internalizing problems of self-identified victims.

## Methods

### Participants and Procedure

Participants were recruited as part of the 6^th^ wave of the Kandinsky Longitudinal Study (KLS), which began in 2010 to identify youth at risk for socio-emotional difficulties (van den Berg et al., 2019). This project was conducted upon request of the school to assess and monitor the social-emotional well-being of their students. The head of school provided parents with a detailed letter describing the study. No parents objected to the participation of their son or daughter. Adolescents were also informed of the details of the study and were asked for active assent at each assessment. No students declined to participate at any stage of the assessment. This procedure was approved by the Institutional Review Board of the Behavourial Science Institute at Radboud University (Protocol Number: ECG2012-2505-038; Project Title: “Sociometry as a method to measure social relationships among children and adolescents”).

Participants were 833 Dutch adolescents in the first three years of secondary school (grades 7-9; 50.2% boys, *M*_age_ = 13.65 years). Twenty-nine students were absent on the day of data collection. Moreover, some students had missing data on the dependent variables and were therefore excluded from the analyses comparing victim types on loneliness (36 missing) and self-esteem (34 missing). This yielded a final sample 768 adolescents (50.4% boys, *M*_age_ = 13.69, *SD* = 1.01) for the analyses of loneliness and 770 for the analyses of self-esteem (50.4% boys, *M*_age_ = 13.69, *SD* = 1.01).

Students with missing data did not differ in gender from students with complete data, χ^2^s > 0.145, *p*s > 0.704. However, students with missing self-reported loneliness had fewer most popular nominations, more least popular nominations, and higher levels of self-reported victimization than students with complete data (*t*s > 1.986, *p*s < 0.047). Students with missing self-esteem also had fewer most popular nominations and more least popular nominations than students with complete data (*ts* > 3.253, *ps* < 0.003).

### Measures

#### Victimization (self-report)

Participants completed an extended version of the Olweus’ Bully-Victim questionnaire (Solberg & Olweus, 2003), which included six items about victimization (e.g., “how often have other students ignored you”). Items were rated on a scale from 1 (*“never”*) to 5 (*“several times a week”*) and averaged to one victimization score (Cronbach’s α = 0.71).

#### Victimization (peer-report)

Participants were asked to nominate “who in your class are bullied by others?”. Classmates’ names were presented in random order between participants, but in the same order across questions within participants. Participants could name an unlimited number of classmates, but not themselves as their name was not presented on the screen. Nominations received were counted for each student and standardized within classrooms.

#### Popularity (peer-report)

Following the same procedure, participants nominated classmates who were “most popular” and “least popular”. For both items, nominations received again were counted for each student and standardized within classrooms.

#### Loneliness (self-report)

Loneliness was assessed with the 12-item peer-related loneliness scale of the Louvain Loneliness Scale for Children and Adolescents (e.g., “I think I have fewer friends than others”; Goossens, 2016). Responses ranged from 0 (*“never”*) to 3 (*“often”*), with higher scores indicating more loneliness and were averaged to one loneliness score (Cronbach’s α = 0.89).

#### Self-Esteem (self-report)

Self-esteem was assessed with the 10-item Rosenberg Self-Esteem scale (e.g., “I can do things as well as most others”; Rosenberg, 1965). Responses ranged from 1 (*“totally disagree”)* to 4 (*“totally agree”*). After reverse coding, higher scores indicated higher self-esteem. An average score across the 10 items was computed for each participant (Cronbach’s α = 0.89).

## Results

### Descriptive Statistics

Means, standard deviations, and correlations among study variables are presented in Table 1. Self- and peer-nominated victimization were positively correlated. Peer-reported, but not self-reported, victimization was negatively associated with popularity. Self- and peer- reported victimization were positively associated with unpopularity and loneliness. Self-reported, but not peer-reported, victimization was negatively associated with self-esteem. Loneliness was negatively associated with being most popular and positively associated with being least popular.

**Table 1:** Means, standard deviations, and correlations for main study variables

	1.	2.	3.	4.	5.	*M*	*SD*
1. Self-reported victimization	–					1.40	0.47
2. Peer-nominated victimization	0.27^***^	–				0.00	0.99
3. Most popular	0.01	−0.23^***^	–			0.00	0.99
4. Least popular	0.13^***^	0.61^***^	−0.41^***^	–		−0.00	0.99
5. Loneliness	0.48^***^	0.28^***^	−0.19^***^	0.28^***^	–	1.36	0.45
6. Self-esteem	−0.32^***^	−0.04	0.04	−0.06	−0.50^***^	3.22	0.54

*n* = 804. 768 participants had data on loneliness and 770 on self-esteem

*p < 0.05; **p < 0.01; ***p < 0.001

### Identifying Victim Types

We conducted a series of latent profile analyses (LPA) using tidyLPA in R (Rosenberg et al., 2018). LPA compares participants on continuous variables to assign them to mutually exclusive groups. We used four variables: self-reported victimization, and the peer nomination scores for victimization, most popular, and least popular. Fit is determined by comparing models on various statistical information criteria, with the Bayesian information criterion (BIC) considered the most accurate indicator of the number of classes (Nylund et al., 2007). Examination of the possible classes revealed that the LPA did not identify distinct groups of victims and non-victims; some classes contained both victims and non-victims. Additional information regarding this LPA for the full sample can be requested from the first author.

To address this problem and to ensure identification of distinct groups, we created a binary variable from self-reported victimization to differentiate between victims and non-victims. Participants were categorized as victims if their combined victimization experience across all forms yielded a frequency of approximately 2 to 3 times a month, which is the recommended and widely used threshold to identify victims (e.g., Solberg & Olweus, 2003). This resulted in approximately 1/3 of the sample (34.8%) being identified as a victim (self-reported victimization = 1) and the remaining 2/3 of the sample being identified as a non-victim (self-reported victimization = 0) which is comparable to prevalence rates from other studies (e.g., Modecki, Minchin, Harbaugh, Guerra, & Runions, 2014). Following this process, separate latent profile analyses were conducted for victims and non-victims using three variables: the peer nomination scores for victim, most popular, and least popular (see Table 2 for full model comparisons).

**Table 2:** Model fit indices for latent profile analyses specifying one to nine latent classes

	LLR	AIC	BIC	ABIC	BLRT
	Self-reported victimization = 1, *n* = 280				
1 Class	1292.18	2596.37	2618.18	2599.15	N/A
2 Classes	1176.81	2373.62	2409.97	2378.26	0.001
**3 Classes**	**967.38**	**1962.75**	**2013.64**	**1969.25**	**0.001**
4 Classes	935.49	1906.99	1972.42	1915.34	0.001
5 Classes	905.41	1854.81	1934.78	1865.02	0.001
6 Classes	879.76	1811.51	1906.02	1823.57	0.001
7 Classes	756.81	1573.63	1682.67	1587.54	0.001
8 Classes	714.88	1497.75	1621.34	1513.52	0.001
9 Classes	703.97	1483.94	1622.06	1501.56	0.003

	Self-reported victimization = 0, *n* = 524				
	LLR	AIC	BIC	ABIC	BLRT
1 Class	2035.96	4083.89	4109.46	4090.42	N/A
2 Classes	1873.83	3767.65	3810.27	3778.53	0.001
**3 Classes**	**1640.02**	**3308.05**	**3367.71**	**3323.27**	**0.001**
4 Classes	1559.63	3155.26	3231.97	3174.84	0.001
5 Classes	1311.04	2666.09	2759.84	2690.01	0.001
6 Classes	1257.11	2566.22	2677.01	2594.48	0.001
7 Classes	1132.15	2324.30	2452.15	2356.92	0.001
8 Classes	1077.86	2223.72	2368.61	2260.68	0.001
9 Classes	1052.98	2181.95	2343.89	2223.27	0.001

BLRT was not available for the one class model. BLRT discontinues once the model fit between *k* and *k –* 1 is not statistically significant.

The bolded values represent the selected model.

*AIC* Akaike Information Criterion, *BIC* Bayesian Information Criterion, *ABIC* Adjusted BIC, *BLRT* Bootstrap Likelihood Ratio Test

For participants who self-reported being victimized (*n* = 280), model fit continued to improve significantly up until nine classes using a bootstrap likelihood ratio test, which tests the model fit between *k*-1 and *k* models (Nylund et al., 2007). In addition to comparison of fit indices, it is recommended to compare the classes that emerge from each model to identify whether they are interpretable and sufficiently large (Nylund-Gibson & Choi, 2018). On this basis, we chose the three-class model. Overall entropy for the three-class model was 0.99, indicating that the three groups were homogenous.

A similar pattern was found for youth who did not self-report any victimization (*n* = 524; see Table 2). This resulted in a total of six victim type groups: three for participants who self-reported being victimized and three for participants who did not self-report victimization.

Table 3 presents the means of the six groups on the three clustering variables. Convergent victims (Group 1; *n* = 31, 3.9%) self-reported being victimized and scored above average on peer-reported victimization. They scored below average on most popular and above average on least popular. Self-identified victims with high popularity (Group 2, *n* = 63, 7.8%) self-reported being victimized but scored below average on peer-reported victimization. They scored above average on most popular and below average on least popular. Self-identified victims with average popularity (Group 3, *n* = 186, 23.1%) also scored below average on peer-reported victimization. However, they scored below average on both most and least popular. Peer-identified victims (Group 4, *n* = 65, 8.1%) did not self-report being victimized but scored above average on peer-reported victimization. This group scored below average on most popular and above average on least popular. Finally, two types of non-victims were identified with varying levels of popularity. Non-victims with high popularity (*n* = 78, 9.7%) did not self-report being victimized and scored below average on peer-reported victimization. They scored above average on most popular and below average on least popular. Non-victims with average popularity (*n* = 381, 47.4%) did not self-report being victimized and scored below average on peer-reported victimization. This group scored below average on both most and least popular.

**Table 3:** Comparison of independent and dependent variables by victim subgroups identified with LPA

	Self-reported victims			Self-reported non-victims		
	Convergent, low popularity (*n* = 31)	Self-identified, high popularity (*n* = 63)	Self-identified, average popularity (*n* = 186)	Peer-identified, low popularity (*n* = 65)	Non-victim, high popularity (*n* = 78)	Non-victim, average popularity (*n* = 381)
Independent variables						
Peer-reported victimization	3.42 (1.17)^a^	−0.37 (0.16)^c^	−0.13 (0.45)^c^	0.81 (1.58)^b^	−0.36 (0.12)^c^	−0.22 (0.51)^c^
Most popular	−0.66 (0.13)^b^	1.88 (0.65)^a^	−0.37 (0.40)^c^	−0.70 (0.17)^b^	1.88 (0.73)^a^	−0.33 (0.44)^c^
Least popular	1.95 (1.54)^a^	−0.58 (0.13)^d^	−0.03 (0.78)^c^	2.08 (0.79)^a^	−0.63 (0.14)^d^	−0.28 (0.43)^c^
Dependent variables						
Loneliness	2.01 (0.07)^a^	1.32 (0.05)^c,d^	1.63 (0.03)^b^	1.43 (0.05)^c^	1.13 (0.04)^e^	1.22 (0.02)^d,e^
Self-esteem	3.02 (0.08)^b^	3.22 (0.05)^a,b^	3.13 (0.03)^b^	3.33 (0.06)^a^	3.34 (0.06)^a^	3.32 (0.03)^a^

Independent variables (*M*s and *SD*s reported) were included in the LPA to identify victim subgroups. Predicted adjusted means and standard errors from the multilevel mixed-effects linear regression analyses predicting the dependent variables are reported. All regression models controlled for gender. Means in the same row that do not share superscripts differ at *p* < 0.05 using Holm-Bonferroni adjusted *p* values for multiple comparisons

### Loneliness and Self-esteem of Victim Types

To examine group differences in loneliness and self-esteem, we conducted multilevel mixed-effects linear regression with maximum likelihood estimation to account for the nested nature of the data with students nested in classrooms. We ran two unconditional models, one for loneliness and one for self-esteem, to assess the amount of variance between and within classrooms. The intraclass correlation coefficient (ICC) was 0.009 for loneliness and 0.017 for self-esteem, indicating that between 0.9% to 1.7% of the variance in the dependent variables was between classrooms.

To test hypotheses regarding group differences, we ran a series of models, changing which victim group served as the reference group. To account for these multiple comparisons, we used the Holm-Bonferroni *p*-value adjustment to assess whether group differences were significant (Abdi, 2010; Holm, 1979). Analyses controlled for gender (0 = boys, 1 = girls) given significant gender differences in victim group membership in the current sample, χ^2^(5, *n* = 804) = 12.233, *p* = 0.032, with more boys being popular non-victims and more girls being non-victims with average popularity than expected. Table 3 presents the adjusted means post model estimation, accounting for gender.

As expected, convergent victims reported significantly more loneliness than all other groups, *p*s < 0.001. Self-identified victims with average popularity had the second highest levels, significantly higher than the remaining groups (*p*s < 0.001). Self-identified victims with high popularity reported significantly less loneliness than self-identified victims with average popularity, *p* < 0.001. Popular self-identified victims reported significantly more loneliness than popular non-victims (*p* = 0.004) but did not differ from average popular non-victims (*p* = 0.059). Peer-identified victims were significantly lonelier than both non-victim groups (*ps* < 0.001). As expected, there was no significant difference in loneliness between the non-victim groups with high and average popularity (*p* = 0.061).

Convergent victims and self-identified victims with average popularity had similar levels of self-esteem (*p* = 0.73), which were significantly lower than peer-identified victims and both non-victim groups (*p*s < 0.002). Popular self-identified victims had lower self-esteem than non-victims with average popularity, but the difference failed to reach the adjusted *p*-value threshold of 0.0056 for significance (*p* = 0.008). In fact, popular self-identified victims did not significantly differ from any other group in self esteem (0.017 < *p*s < 0.073). There were no significant differences in self-esteem between popular non-victims, average popular non-victims, and peer-identified victims (*p*s < 0.958).

### Additional Analyses

In the primary analyses, self-reported victimization was treated as a binary variable because the full-sample LPA was unable to identify distinct groups of victims and non-victims with the continuous self-reported victimization variable. Accordingly, the primary analyses did not differentiate the extent to which youth self-reported victimization. As an exploratory analysis, we also identified groups of victims and non-victims using cut-off scores (> 0.5 *SD* above the mean) for self- and peer-reported victimization. Consistent with past research (e.g., Dawes et al., 2017, 2019; Scholte et al., 2013), we identified convergent victims (*n* = 43), self-identified victims (*n* = 154), peer-identified victims (*n* = 57), and non-victims (*n* = 550). There was more variance in popularity for self-identified victims and non-victims (*SDs* = 1.07 and 1.01, respectively) than for convergent and peer-identified victims (*SD*s = 0.22). Of the 154 self-identified victims, 44 were high in popularity (>*M* + 0.5 *SD*). Of the 550 non-victims, 134 were high in popularity. This resulted in six victim types (see Table 4): convergent (5.3%), self-identified with high popularity (5.5%), self-identified with average popularity (13.7%), peer-identified (7.1%), popular non-victims (16.7%), and average popular non-victims (51.7%).

**Table 4:** Comparison of independent and dependent variables by victim subgroups identified with cut-off scores

	Self-reported victims			Self-reported non-victims		
	Convergent, low popularity (*n* = 43)	Self-identified, high popularity (*n* = 44)	Self-identified, average popularity (*n* = 110)	Peer-identified, low popularity (*n* = 57)	Non-victim, high popularity (*n* = 134)	Non-victim, average popularity (*n* = 416)
Independent variables						
Self-reported victimization	2.32 (0.67)^a^	2.07 (0.43)^b^	1.98 (0.36)^b^	1.24 (0.16)^c^	1.20 (0.19)^c^	1.17 (0.17)^c^
Peer nominated victimization	2.61 (1.58)^a^	−0.35 (0.18)^c^	−0.24 (0.23)^c^	0.82 (1.39)^b^	−0.35 (0.12)^c^	−0.30 (0.19)^c^
Most popular	−0.60 (0.22)^b,d^	1.69 (0.73)^a^	−0.42 (0.34)^b^	−0.65 (0.22)^d^	1.61 (0.81)^a^	−0.43 (0.33)^b,c^
Dependent variables						
Loneliness	1.92 (0.06)^a^	1.45 (0.06)^c^	1.71 (0.04)^b^	1.46 (0.05)^c^	1.17 (0.03)^d^	1.26 (0.02)^d^
Self-esteem	2.94 (0.08)^b^	3.00 (0.07)^b^	2.90 (0.05)^b^	3.30 (0.07)^a^	3.29 (0.04)^a^	3.32 (0.03)^a^

Independent variables (*M*s and *SD*s reported) were included in the LPA to identify victim subgroups. Predicted adjusted means and standard errors from the multilevel mixed-effects linear regression analyses predicting the dependent variables are reported. All regression models controlled for gender. Means in the same row that do not share superscripts differ at *p* < 0.05 using Holm-Bonferroni adjusted *p* values for multiple comparisons

We again conducted two separate multilevel mixed-effects linear regression for loneliness and self-esteem. We still controlled for gender, even though the results indicated no significant gender differences in group membership, χ^2^(5, *n* = 804) = 8.472, *p* = 0.132. We accounted for multiple comparisons with the Holm-Bonferroni *p*-value adjustment (Abdi, 2010; Holm, 1979). Table 4 presents the adjusted means post model estimation. The results were very similar to the primary analyses.

Convergent victims had significantly higher levels of loneliness than all other groups (*p*s < 0.005). Self-identified victims with average popularity status reported the second highest levels of loneliness, significantly more than the other victim and non-victim groups (*p*s < 0.001). Peer-identified victims had the third highest level, more than the non-victim groups (*p*s < 0.001). There was no significant difference in loneliness between peer-identified and self-identified victims with high popularity (*p* = 0.879). The non-victim groups with high and average popularity were less lonely than popular self-identified victims (*p*s < 0.002) and did not differ from one another (*p* = 0.025; Holm-Bonferroni adjusted *p*-value for comparison *p* < 0.025).

For self-esteem, convergent victims, popular self-identified victims, and self-identified victims with average popularity had significantly lower levels of self-esteem than the other groups (*p*s < 0.002) but did not significantly differ from one another (*p*s > 0.25). The two non-victim groups and peer-identified victim group did not differ significantly in self-esteem (*p*s > 0.627).

## Discussion

Given the serious and long-term adjustment difficulties associated with victimization (McDougall & Vaillancourt, 2015), it is essential to have a comprehensive understanding of who is at risk. This study addressed two major challenges to successfully identifying victimized youth. One challenge is the relatively low concordance between self- and peer- reports of victimization, the most common ways of assessing victimization. A second challenge is that the majority of research has assumed that victimized youth are socially marginalized with low status, whereas growing evidence supports that high-status youth are also targets of aggression (see Dawes & Malamut, 2018 for a review). With these challenges in mind, the current study first used person-centered analyses of self- and peer-reports of victimization and peer-reports of popularity status to identify victim types with varying levels of popularity. Second, differences in loneliness and self-esteem across the resulting victim types were examined to test how popular victims compare to other victims and non-victims.

### Heterogeneity Across and Within Victim Types

As predicted, there was heterogeneity in popularity across and within the victim types. Using both latent profile analysis and cut-off scores, two types of self-identified victim and non-victim groups were identified, in addition to convergent and peer-identified victims, resulting in a total of six victim groups. These person-centered analyses revealed a subset of self-identified victims with high popularity. Because they do not fit the typical profile of victims, self-identified victims with high popularity may be overlooked – not just by peers, but by teachers as well. Indeed, there is evidence that victimized youth feel that teachers do not understand the severity of bullying when youth do not fit the idea of a typical victim (Bjereld et al., 2019). This is concerning, as recent evidence suggests that popular youth who self-report experiencing high levels of victimization show elevated levels of aggression (Malamut et al., 2020). By overlooking popular victims, researchers may be missing an important factor contributing to the perpetuation of aggression in the peer network. Whereas there is preliminary evidence from variable-centered analyses that being victimized and having high status is related to low adjustment (Faris & Felmlee, 2014; Malamut et al., 2020), person-centered analyses were needed to identify heterogeneity in the associations between self- and peer- reported victimization, popularity status, and adjustment. This study marks the first attempt to disentangle these associations and therefore adds to the existing literature by uncovering different subgroups of victims, including self-reported victims high in popularity, as well as their related internalizing problems.

Regardless of classification method (LPA or cut-off scores), convergent victims and self-identified victims with average status were the most lonely (convergent victims even more so than self-identified victims with average status) and had the lowest self-esteem. Non-victims were the least lonely and had the highest self-esteem. Peer-identified victims may be somewhat protected against the internalizing symptoms that are typically associated with victimization, as they did not differ in self-esteem from non-victims. This suggests that a victim reputation does not necessarily prompt the same cognitive processes as perceiving oneself as victimized (e.g., victim schema, self-blame; Graham & Juvonen, 1998) that tend to be related to low self-esteem. However, peer-identified victims were significantly lonelier than both types of non-victims, which is logical given their increased likelihood for being rejected by peers and having fewer friends (Scholte et al., 2013). Teachers may need to support peer-identified victims by promoting positive relationships with prosocial friends to alleviate their loneliness (Farmer et al., 2021).

Of particular interest to the current study were self-identified victims with high popularity. We formulated two alternative hypotheses in which popularity would either amplify the negative consequences of being victimized or serve as a buffer. Partial support was found for the second hypothesis when comparing groups identified via both methods (i.e., LPA and cut-off scores) on loneliness but not for self-esteem. Self-identified victims with high popularity were less lonely than self-identified victims with average popularity, suggesting that their popularity protected them against loneliness. However, the two self-identified victim groups did not differ in self-esteem, suggesting that popularity does not buffer victims against low self-esteem. Although the evidence suggests that popularity may protect youth from some of the internalizing problems typically experienced by self-identified victims, popular self-identified victims still displayed adjustment difficulties compared to other groups, indicating that the buffering effect of popularity has limits. Popular self-identified victims reported more loneliness than popular non-victims (based on LPA and cut-off scores) and average popular non-victims (based on cut-off scores). In addition, self-identified victims with high popularity had similar levels of loneliness as peer-identified victims (based on both methods). As for self-esteem, popular victims reported lower levels than both popular and average popular non-victims (based on cut-off scores). Collectively, the evidence indicates that popular victims are at risk for greater internalizing problems than their non-victimized peers.

The results for differences in self-esteem when comparing victims based on cut-off scores reveals a tale of two experiences: those with self-reported victimization (convergent and self-identified victim groups) had lower self-esteem than those who did not self-report being victimized (peer-identified victims and non-victim groups). This suggests that adolescents’ own subjective, lived experience with victimization may be particularly damaging, more than having a victim reputation. This underscores the pressing need to listen to and support youth who disclose that they are victimized, even when they do not look like the typical victim (i.e., popular self-identified victims).

### Methodological Considerations

The results have two important implications for how victims are identified. First, it is important to address that popular victims were identified with self-reports and not peer-reports. This is line with recent discussions of the methods to identify high-status victims (Dawes & Malamut, 2018). It is also consistent with the previously found negative correlation between popularity and peer-reported victimization (e.g., Sandstrom & Cillessen, 2006). High-status victims may be missed when identifying victims via peer nominations of general bullying, rather than more specific forms (e.g., rumor spreading; Malamut et al., 2018). In the past, the discrepancy between self- and peer-reports of victimization has been considered a product of biased perceptions (e.g., Rosen et al., 2007). This may be true for some youth, but our results suggest that the discordance may also be due to a subset of youth who are victimized but also popular – a phenomenon that is counter to the narrative that victims are low-status and disempowered.

Second, the prevalence of the victim groups varied slightly depending on the analytic approach. With both LPA and cut-off scores, the prevalence of both convergent and peer-identified victims was low. However, the prevalence of self-identified victims varied somewhat across methods. The LPA classified 31.0% of students as self-identified victims, of which 25.3% had high levels of popularity. The cut-off score method identified only 19.2% of all students as self-identified victims, of which 28.6% were high in popularity. The LPA identified victimized youth who would not have been considered as such by the cut-off analyses. Although this has implications for how victims are identified, the group profiles of internalizing problems were largely similar across methods. Notably, popular self-identified victims were identified even when using cut-off scores to identify victimized youth with the highest levels of self-reported victimization. This demonstrates not only that popular youth can be victimized, but also that some popular youth are among those reporting the highest levels of victimization.

### Strengths, Limitations and Future Directions

This study built on prior research by using a person-oriented approach to examine the associations between popularity and victimization and the heterogeneity of popularity among victims. In addition, the current study used and compared an advanced classification method (LPA) with a traditional cut-off scores approach, thereby making a methodological contribution. Moreover, the nested nature of the data was accounted for, which corrects for any dependencies within classrooms.

Despite these strengths, this study also had some limitations. First, the correlational nature of the study prevents conclusions about the direction of effects between victimization and loneliness and self-esteem. There is evidence from longitudinal research that internalizing problems are both predictors and outcomes of peer victimization (Reijntjes et al., 2010). The main goals of the current study were not longitudinal in nature; we wanted to identify a subgroup of popular victims and examine how their internalizing problems compared to other victim types. Future research could examine changes in victims’ loneliness and self-esteem over time and whether such changes are related to concomitant changes in youth’s popularity status as well.

Second, the self-reports of victimization, loneliness, and self-esteem shared method variance. Although it is typically preferred to include other informants (e.g., peers, teachers), the key focus of this study was on understanding victim groups’ feelings of loneliness and self-esteem, which are most reliably measured through self-report. This is particularly relevant for popular victims who may go unnoticed by teachers and peers because they do not look like typical victims (i.e., halo effect; Marucci et al., 2021). It is unlikely that teachers and peers would accurately identify internalizing problems in popular victims, especially if they are unlikely to recognize their experiences of victimization.

Third, we did not address if victimized youth were also perpetrators. It has been argued that victimization and aggression form a continuum (e.g., Graham et al., 2006; Solberg & Olweus, 2003), and victimization can indeed be a predictor of later aggression (e.g., Malamut & Salmivalli, 2021). Thus, future research should address which victims are also targeting their peers. One question for future research is whether popular self-identified victims are also perpetrators who are playing the “victim card” to justify their own perpetration. However, the results of this study indicate that high-status victims *do* experience some distress relative to other youth. Future research should investigate whether they are also aggressors, with implications for interventions efforts.

## Conclusion

There is still much that is unknown regarding victims with varying levels of status, particularly victims with high levels of popularity. Given the relative paucity of research on the adjustment of popular victims, information about their internalizing indices was critically needed. To address this gap, the current study used person-oriented analyses to identify heterogeneity in status across and within victim types. The results supported growing evidence that victimization experiences are not limited to low-status youth (see, for a review, Dawes & Malamut, 2018). A sizeable group of popular youth who reported high levels of victimization were identified. Further, differences were found in loneliness and self-esteem between victim groups varying in popularity. The mere existence of this group challenges long-standing assumptions about victimized youth (i.e., that they are all socially marginalized) and assumptions that popularity insulates youth from being targets of their peers’ aggression or the negative consequences associated with victimization. This has important implications as adults (e.g., teachers, parents) may not recognize or even believe the victimization experiences of some popular youth. The results suggest that popularity should be considered alongside victimization reports to provide nuanced information to better identify victims of peer aggression, particular youths who do not fit the typical victim profile. This research direction is critical for a comprehensive understanding of victimization dynamics among peers and intervention efforts to support all types of victims.
